# High-Speed Terahertz Waveform Measurement for Intense Terahertz Light Using 100-kHz Yb-Doped Fiber Laser

**DOI:** 10.3390/s18061936

**Published:** 2018-06-14

**Authors:** Masaaki Tsubouchi, Keisuke Nagashima

**Affiliations:** Kansai Photon Science Institute (KPSI), National Institutes for Quantum and Radiological Science and Technology (QST), 8-1-7 Umimedai, Kizugawa, Kyoto 619-0215, Japan; nagashima.keisuke@qst.go.jp

**Keywords:** terahertz, THz spectroscopy, THz imaging, high-speed measurement

## Abstract

We demonstrate a high-speed terahertz (THz) waveform measurement system for intense THz light with a scan rate of 100 Hz. To realize the high scan rate, a loudspeaker vibrating at 50 Hz is employed to scan the delay time between THz light and electro-optic sampling light. Because the fast scan system requires a high data sampling rate, we develop an Yb-doped fiber laser with a repetition rate of 100 kHz optimized for effective THz light generation with the output electric field of 1 kV/cm. The present system drastically reduces the measurement time of the THz waveform from several minutes to 10 ms.

## 1. Introduction

Terahertz (THz) light is used for the inspection of semiconductor devices and biological tissue and for security and other applications [1,2,3,4,5,6,7,8]. THz light has low photon energy, which allows nondestructive and noninvasive probes to be used for these inspections without influencing the molecular structure and electronic properties. THz time-domain spectroscopy (THz-TDS) is widely used as a sensitive and accurate method to obtain both THz waveforms and its Fourier transform spectra of THz light. Using this advantage of THz-TDS, a time-of-flight THz tomography has been developed for measurement of layer structures near the surface [9]. In addition, a two-dimensional (2D) THz hyperspectral imaging based on THz-TDS has been demonstrated for the inspection of drugs and food items, for investigation of ancient arts, and for medical diagnostics [10,11,12,13] and has been commercially available.

To establish THz imaging as a practical tool, the acquisition speed of THz imaging systems with THz-TDS must be increased by two to three orders of magnitude, as suggested in the roadmap of the THz science and technology given in Reference [14]. The slow acquisition speed of THz hyperspectral imaging is mainly because the time-domain scan needs to obtain a waveform in THz-TDS in addition to the spatial 2D scan. In the conventional THz-TDS setup, a linear mechanical delay stage is employed to scan the time delay between the THz and the sampling pulses. Because the stage is decelerated and accelerated in each measuring step of the THz electric field, the acquisition time of the entire THz waveform easily exceeds several minutes. Therefore, we have to establish a method of high-speed THz waveform measurement for practical THz imaging.

In THz-TDS systems that use weak femtosecond pump light with repetition rates of more than 10 MHz, high-speed THz waveform measurements have already been demonstrated. The mechanically rotating mirrors have realized scan rates of 400 Hz [15,16]. Some studies have proposed electro-optical control of time delay, rather than the mechanical delay scan, for high-speed measurements with kHz scan rates. These include asynchronous optical sampling [17,18], optical sampling by cavity tuning [19], and electrically controlled optical sampling [20,21]. Two mode-locked lasers with a fixed difference of repetition rates introduce a linear sweep of time-delay between the pump and probe pulses. The scan rate of the THz waveform is equal to the difference of the repetition rates, which is in the range of 0.1 to 10 kHz.

Intense THz light has been obtained in table-top laser systems. However, the low repetition rate of the amplified laser system limits the scan rate to below 1 Hz. A high scan rate in THz-TDS with intense THz light can extend THz applications. Photo-induced dynamics of semiconductor, polymer, biological tissue, etc. can be effectively measured by optical pump-THz probe time-resolved spectroscopy with high-speed measurements. The THz imaging system ensures that high speed simultaneous THz waveform measurements in 1D or 2D space are feasible with the intense THz light. To realize high-speed THz waveform measurements with intense THz light, single-shot measurements of the waveform without time-domain scan have been proposed. These are based on mapping of the time-domain data onto the spatial position of the electro-optic (EO) crystal by noncollinear geometry for the THz and probe light [22] or a reflective echelon mirror [23], and mapping onto the frequency spectrum by a chirped probe pulse [24,25]. The scan rate of these promising methods depends on the laser’s repetition rate and the data accumulation time and is typically 1 to 10 Hz.

In this study, we have developed an advanced method to achieve high-speed measurement of the THz waveform with intense THz light. Our method is based on the time-domain scan with high-speed vibration of a translational stage and achieves a scan rate of 100 Hz with easy operation. In previous single-shot measurements, the waveform had to be transformed from the observed spatial mapping or spectrum with appropriate calibration. The parameters used in the calibration are sensitive to the optical alignment and, therefore, must be determined in each experiment. Furthermore, it is difficult to linearly map the time-domain data onto the spatial or spectral region across a wide range (typically less than 10 ps). By contrast, our method does not require complicated calibration because the THz waveform is directly measured by the time-domain scan. The time range of the scan is only limited by the maximum vibration amplitude of the translation stage.

This paper is organized as follows: First, we describe experimental details of high-speed THz waveform measurement in Section 2. To realize the high-speed measurement of the THz waveform with intense THz light and to apply it to practical study such as the THz hyperspectral imaging, we thoroughly design the total system and optimize well-known technical components, e.g., an Yb-doped fiber laser, a loudspeaker scanner, a THz-TDS system, and so on. Next, we demonstrate and evaluate our system in Section 3. We conclude with future perspectives in Section 4.

## 2. Experimental Details

We constructed a system based on a high-speed time-domain scan to obtain THz waveforms with a high scan rate. To scan a time delay between the THz and EO probe pulses quickly, we used a loudspeaker as a high-speed scanner that has been used in autocorrelators to measure the pulse width of ultrashort laser pulses [26]. This method has been introduced for THz waveform measurement in a photoconductive antenna system [27]. In our system, the achieved scan rate of the waveform was 100 Hz with the speaker’s vibration at 50 Hz; two waveforms were obtained in a single round trip. The mechanically rotating mirror was also one of the candidates for the high-speed time-domain scan for intense THz light. However, when we used the rotating mirror to reflect the intense pump light, we had to process a high-reflection (HR) multilayer coating on the curved metal surface of the rotating mirror, which was technically very difficult. On the other hand, we could use the bare metal surface as the rotating mirror for the weak EO probe light. But the rotating mirror was not proper for the scan of the probe light because EO detection is very sensitive to the beam pointing of the probe light on the EO crystal. As a result, we adopted the loudspeaker as a high-speed scanner instead of the rotating mirror.

When the scanning system is operated at 100 Hz, the repetition rate of THz pulse generation has to be increased also. For example, the THz generation system with a 1 kHz repetition rate can plot only 10 points in a single waveform, which is insufficient for practical THz waveform measurement. Therefore, we constructed a laser system with a 100 kHz repetition rate in which 1000 data points can be plotted in a waveform.

### 2.1. THz Light Generation

**Figure 1:**
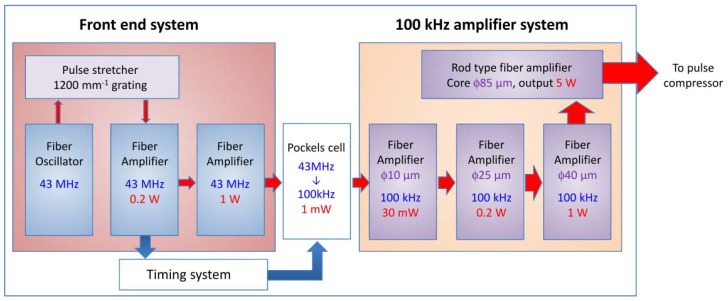
Schematic of the Yb-doped fiber laser system.

First, we designed the effective THz generation scheme with a 100 kHz repetition rate. Currently, an optical rectification in LiNbO_3_ (LN) crystal with a titled pulse front technique is widely used to generate intense THz light [28,29,30]. Fülöp and co-workers calculated the THz generation process in this method and suggested that the THz generation efficiency was maximized for the transform-limited (TL) pump pulse at the pulse width of 300–400 fs [31]. The Yb-doped fiber laser is one of the most promising systems for effective THz light generation with a 350 fs pulse-width and a 100 kHz repetition rate. Figure 1 shows the schematic of the Yb-doped fiber laser system constructed in this study. A mode-locked fiber oscillator operated at a center wavelength of 1040 nm and a frequency of 43 MHz was employed as a seed laser. The stretched seed laser was amplified to 1 W in two Yb-doped fibers pumped by a 976-nm laser diode. After the second amplifier, 100 kHz pulses with 1 mW were picked from 43 MHz by a Pockels cell and amplified again in four fiber amplifiers to 5 W. The amplified 100 kHz pulses were sent to the pulse compressor and compressed to the TL pulses with a pulse energy of 28 µJ and a pulse width of 320 fs for THz light generation. As mentioned before, the amplification process was divided into six stages to suppress amplified spontaneous emission and to prevent damage to the fibers. The amplified laser power after the compressor increased linearly with increasing diode current in the final amplifier, reaching a maximum value of 10 W (100 μJ/pulse). However, when the output energy was larger than 28 μJ, the amplified pulse could not be compressed to the TL pulse at the compressor due to higher-order dispersion caused by self-phase modulation.

The near-infrared (NIR) pump light was delivered to the THz generation system based on the “contact grating setup”, which was first proposed by Pálfalvi and co-workers [32] and was realized in our previous studies [33,34,35]. In the contact grating setup, the diffraction grating was placed in contact with the input surface of the LN substrate, which resulted in a drastic downsizing of the THz generation system compared with the conventional pulse front tilting method. Therefore, the generation system with the contact grating setup is promising for industrial applications. The contact grating device was fabricated on a 1.3 moL% Mg-doped stoichiometric LN (Mg-sLN) substrate with dimensions 16 mm (Z) × 20 mm (Y) × 2.2 mm (X), where X, Y, and Z are the crystal axes. The grooves were etched parallel to the Z-axis with an effective area of 10 mm (Z) × 14 mm (Y). Details of the device are described in our previous paper [33,34,35].

### 2.2. THz Waveform Measurement

**Figure 2:**
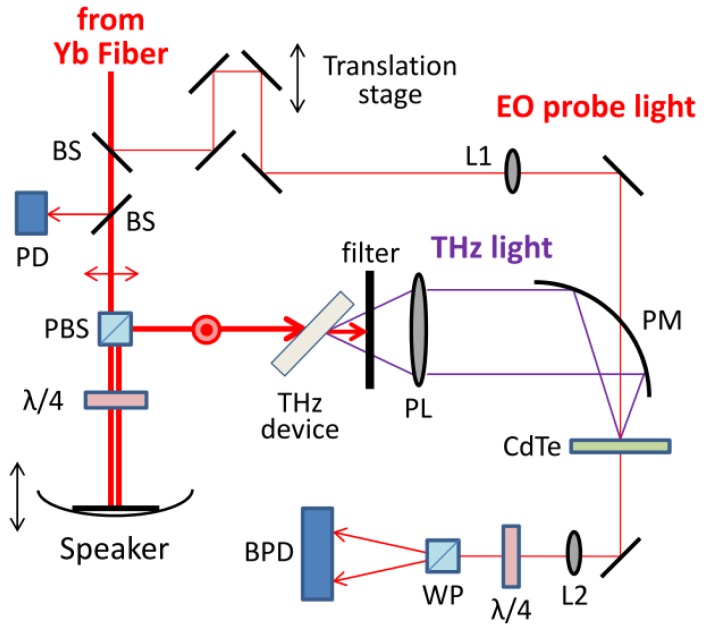
Schematic diagram of the THz generation and detection system. BS: beam splitter; PD: fast photodiode for 100-kHz laser trigger; PBS: polarizing beam splitter; λ/4: quarter wave plate; L1 and L2: BK7 lenses with focal lengths of 250 and 150 mm, respectively; PL: plastic lens for THz light with a focal length of 50 mm; PM: off-axis parabolic gold-coated mirror with a centered hole 2 mm in diameter and a focal length of 50 mm; WP: Wollaston prism; BPD: balanced amplified photodetector.

Figure 2 shows the schematic diagram of the THz generation and detection system. The output NIR pulse from the Yb-doped fiber laser was split into two parts for THz light generation and EO detection. The former part was passed through the polarizing beam splitter (PBS) and reflected by the mirror installed on the vibrating membrane of the speaker. To pick up the reflected light by PBS, the quarter wave plate was placed between PBS and the speaker. The reflected light pumps the contact grating device to generate THz light. Both the pump and THz light were s-polarized to the device. The THz light was separated from the pump light by a plastic plate with a HR coating for the NIR light and a black polypropylene film. The THz light was collimated by an aspheric plastic lens and focused to a detection point by a gold-coated off-axis parabolic mirror. At the detection point, the THz electric field was observed by EO sampling in a 3-mm-thick CdTe crystal. The EO probe light with vertical polarization was reflected by the mirror pair on the stepping motor translation stage and focused on the CdTe crystal. The polarization rotation due to the EO effect was measured by a balanced detection system with a balanced amplified photodetector.

**Figure 3:**
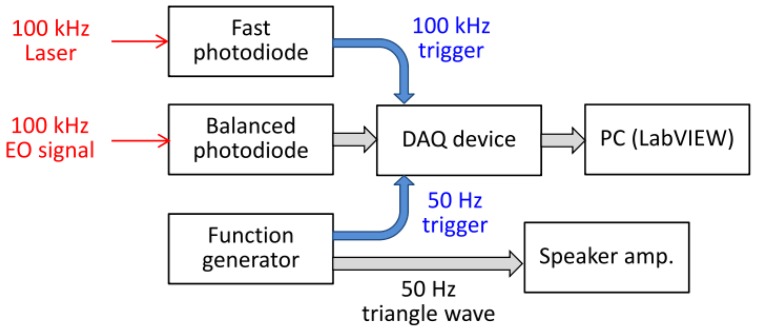
Block diagram of the data acquisition system.

The optical delay between the THz and EO probe light was first adjusted by the stepping motor translational stage and then scanned by the speaker’s vibration. The appropriate speaker was chosen so that the amplitude of the membrane motion ensures a linear with respect to an input voltage. Figure 3 shows the block diagram of the data acquisition system. The Yb-fiber laser generated the 100 kHz clock to the system. The analog EO signal from the balanced detector was recorded via a data acquisition (DAQ) device synchronized with the 100-kHz laser trigger. The speaker vibration was controlled by a function generator, which provided a triangular wave at frequency of 50 Hz. The measurement of the THz waveforms was triggered by 50 Hz TTL signal from the function generator and achieved a scan rate of 100 Hz as mentioned before.

## 3. Results and Discussion

**Figure 4:**
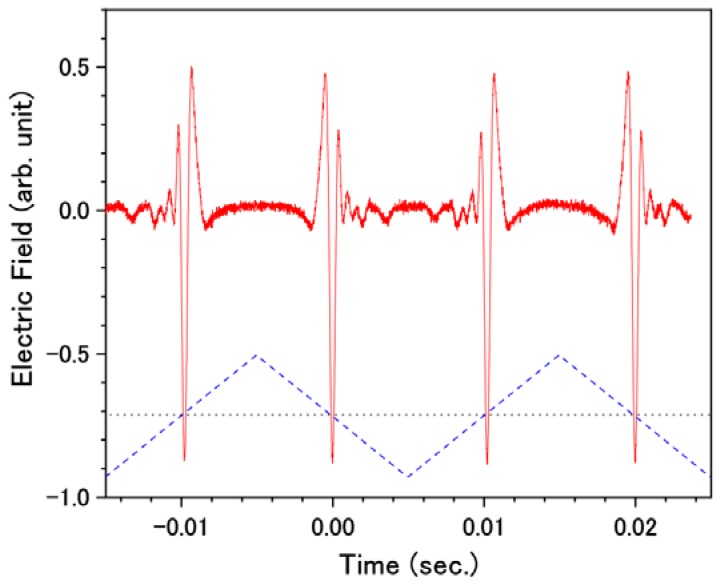
THz waveforms obtained by the data acquisition system described in Figure 2 and Figure 3. The horizontal axis shows the time at which the data was recorded by data acquisition (DAQ) device. The red solid line shows the observed waveform. The blue dashed line indicates the vibration of the speaker membrane around the zero-time delay between the electro-optic (EO) probe and THz light (shown as the black dotted line).

Figure 4 shows the THz waveforms obtained by the data acquisition system described in Figure 2 and Figure 3. The horizontal axis shows the time at which the data was recorded by the DAQ device. The sequence of the THz waveforms was successfully obtained without averaging. Data acquisition was synchronized with a 100-kHz laser trigger and, therefore, the data sampling interval in the waveform was 10 μs. The triangular wave applied to the speaker system is indicated in the Figure 4 as the blue dashed line. The stepping motor translation stage was adjusted so that the THz electric field took the maximum value at the zero-vibration amplitude of the speaker. When a positive voltage was applied to the speaker, the EO probe pulse arrived before the pump pulse at the EO sampling point and vice versa. Within the single period of the triangular wave, a pair of THz waveforms was observed.

**Figure 5:**
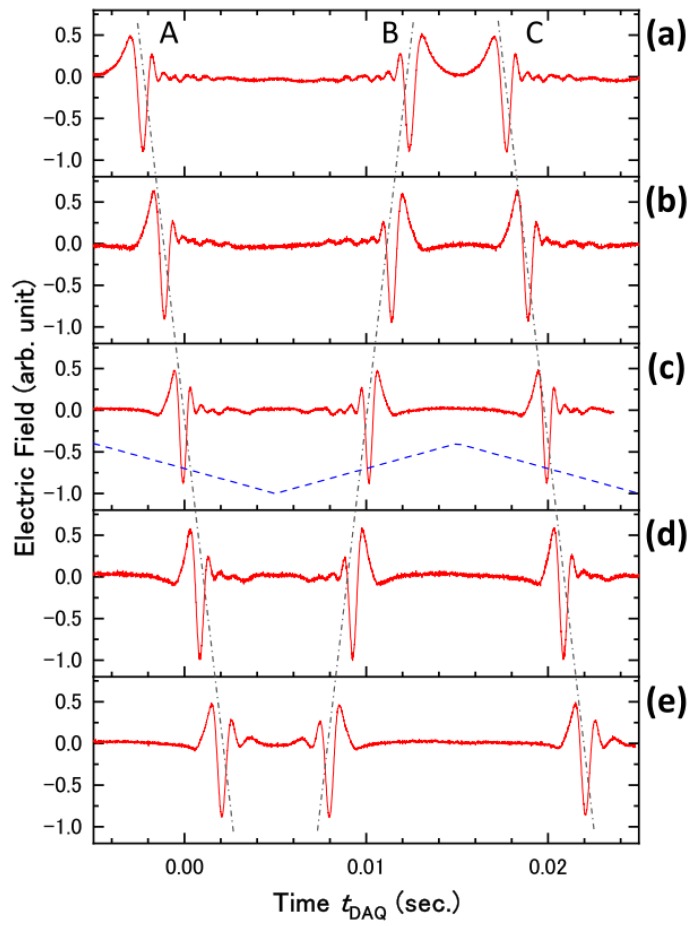
THz waveform observed at the position of the stepping motor translational stage, (**a**) *X* = −500 μm; (**b**) −250 μm; (**c**) 0 μm; (**d**) 250 μm; and (**e**) 500 μm. The red solid line shows the observed waveform. The blue dashed line indicates the vibration of the speaker membrane.

**Figure 6:**
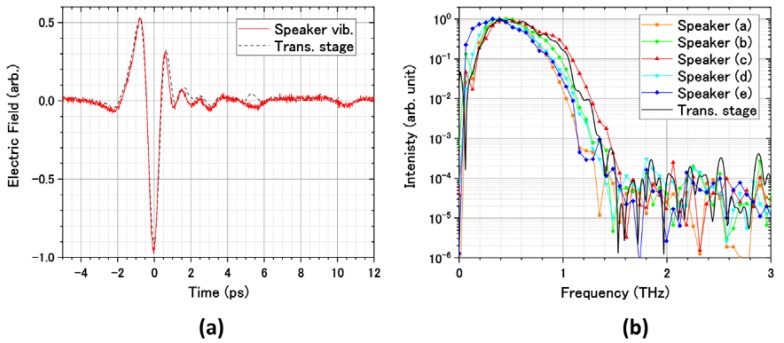
(**a**) THz waveforms and (**b**) its Fourier transformed spectra obtained by using the stepping motor translational stage and the vibrating speaker. The “speaker (a) to (e)” indicates the spectrum obtained from the waveform shown in Figure 5a–e, respectively.

To calibrate the delay time of the EO probe pulse from the pump pulse, we measured the THz waveforms at several positions of the stepping motor translation stage as shown in Figure 5. Three series of the THz waveform—A, B, and C—are shown in Figure 5. The time interval between series A and C was always 20 ms, which was identical to the period of the speaker’s vibration. The time when series A appeared, $t_{DAQ}$, linearly shifted to the later time when increasing the position of the translational stage, *X*: $t_{DAQ}=aX$, where a is the proportionality constant and we define as $t_{DAQ}=0$ at *X* = 0. This result indicates that the displacement of the speaker membrane is proportional to the amplitude of the triangular wave. The delay time t of the EO probe pulse from the pump pulse was related to the position of the translational stage at the specific $t_{DAQ}$ as $t=2X/c_{0}$, where $c_{0}$ is the speed of light. Therefore, the delay time t can be calibrated as $t=2t_{DAQ}/ac_{0}$. In Figure 6a, the calibrated waveform is shown as the solid line and is compared with the waveform measured by the stepping motor translation stage. There is good agreement between waveforms. The THz waveform consists of 1000 data points in the time range of 15 ps with 15 fs intervals. The single waveform with the time range of 15 ps was measured in the measurement time of 10 ms, which was 10^4^ times shorter than that required for the scan by the stepping motor translation stage. By employing pump light with a pulse energy of 13 μJ, the maximum THz electric field of 0.96 kV/cm was obtained. Figure 6b shows the Fourier transformed spectra. The peak frequency and the bandwidth were 0.6 THz and 1.5 THz, respectively. It can be noticed that the waveforms are slightly distorted near the turning points of the membrane vibration in the loudspeaker, as seen in Figure 5a,e. This is due to the lack of linearity in the amplitude of the membrane motion with respect to the applied voltage at the turning points. To understand the distortion of the waveforms quantitatively, we calculated the Fourier transformed spectra from the waveforms shown in Figure 5a–e and compared these spectra with the reference spectrum obtained by conventional translational stage. The spectrum (c) in which the THz electric field took the maximum value at the zero-vibration amplitude of the speaker was almost identical to the reference. However, in the spectra (a) and (e), the discrepancy due to the lack of linearity in the membrane motion can be seen in the high and low frequency regions.

**Figure 7:**
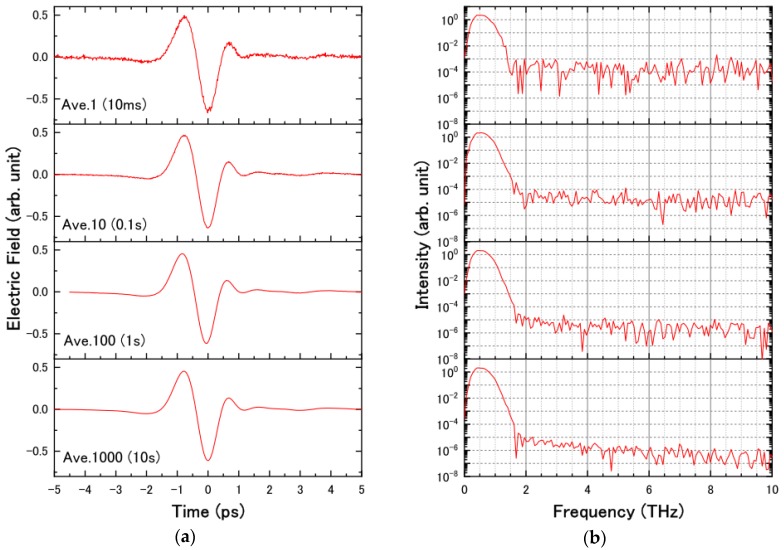
(**a**) THz waveforms and (**b**) Fourier transformed spectra measured by changing the cumulative number.

In the presented study, we have several important parameters describing the apparatus: the scan rate, the frequency resolution, and the signal-to-noise ratio (SNR). The frequency resolution depends on the scanning time range, which is closely related to the scan rate. In our system, we supplied the triangular wave with a frequency of 50 Hz to the speaker and scanned the waveform in the time range of 15 ps. In principle, the scan rate of the THz waveform increases with increasing vibration frequency of the speaker. However, the allowed maximum vibration amplitude of the speaker at high frequencies is too small to scan enough time range in the THz waveform. This mechanical threshold for the speaker limits the scan rate of the waveforms. To discuss the SNR in our apparatus, Figure 7a,b show the THz waveforms and the Fourier transformed spectra measured by changing the cumulative number, respectively. The SNR of the spectrum was 10^4^ even without averaging and went up to 10^6^ with 1 s (100 scans) averaging. As shown in Figure 7, our system realizes the high-precision measurement of the THz waveform within 1 s, which drastically reduces the data acquisition time for the practical applications of the THz-TDS with intense THz light, for example, optical pump-THz probe experiments, time-of-flight tomography, and THz imaging.

## 4. Summary and Perspectives

In this study, high-speed THz waveform measurement with intense THz light has been demonstrated with a scan rate of 100 Hz. To obtain high-speed measurement, we employed a loudspeaker as the high-speed scanner for the delay time of the EO probe light with respect to the THz light, a 100-kHz Yb-doped fiber laser system for high-speed data acquisition, and a contact grating device for THz light generation.

One of the advantages of THz-TDS is that the carrier-envelope phase-locked few-cycle THz pulse is observed directly. Therefore, it is easily applied to time-resolved spectroscopy (e.g., optical pump-THz probe spectroscopy), time-of-flight THz tomography, and THz hyperspectral imaging. The hyperspectra include the spectral information in each pixel distributed in 2D space. Therefore, 3D time-space scanning is required in the conventional THz-TDS with weak THz light. When we apply our fast measurement system with intense THz light, we measure the spectra in pixels of 1D line or 2D plane simultaneously. This is expected to drastically reduce the measurement time of the hyperspectral image from 1 h to 1 s. Our high-speed THz waveform measurement system will accelerate these applications greatly and promote the industrial application of THz science.
